# Emerging Trends and Hot Spots of Electrical Impedance Tomography Applications in Clinical Lung Monitoring

**DOI:** 10.3389/fmed.2021.813640

**Published:** 2022-01-31

**Authors:** Zhe Li, Shaojie Qin, Chen Chen, Shuya Mei, Yulong Yao, Zhanqi Zhao, Wen Li, Yuxiao Deng, Yuan Gao

**Affiliations:** ^1^Department of Critical Care Medicine, Renji Hospital, School of Medicine, Shanghai Jiao Tong University, Shanghai, China; ^2^Department of Biomedical Engineering, Fourth Military Medical University, Xi'an, China; ^3^Institute of Technical Medicine, Furtwangen University, Villingen-Schwenningen, Germany

**Keywords:** bibliometric analysis, EIT, lung, ARDS, hotspot

## Abstract

**Objective:**

This study explores the emerging trends and hot topics concerning applications on electrical impedance tomography (EIT) in clinical lung monitoring.

**Methods:**

Publications on EIT applications in clinical lung monitoring in 2001–2021 were extracted from the Web of Science Core Collection (WoSCC). The search strategy was “electrical impedance tomography” and “lung.” CiteSpace, a VOS viewer was used to study the citation characteristics, cooperation, and keyword co-occurrence. Moreover, co-cited reference clustering, structural variation analysis (SVA), and future research trends were presented.

**Results:**

Six hundred and thirty-six publications were included for the final analysis. The global annual publications on clinical lung monitoring gradually increased in the last two decades. Germany contributes 32.2% of total global publications. University Medical Center Schleswig-Holstein (84 publications, cited frequency 2,205), *Physiological Measurement* (105 publications, cited frequency 2,056), and Inéz Frerichs (116 articles, cited frequency 3,609) were the institution, journal, and author with the largest number of article citations in the research field. “Electrical impedance tomography” (occurrences, 304), “mechanical ventilation” (occurrences, 99), and “acute respiratory distress syndrome” (occurrences, 67) were the top most three frequent keywords, “noninvasive monitoring” (Avg, pub, year: 2008.17), and “extracorporeal membrane oxygenation” (Avg, pub, year: 2019.60) were the earliest and latest keywords. The keywords “electrical impedance tomography” (strength 7.88) and co-cited reference “Frerichs I, 2017, THORAX” (strength 47.45) had the highest burst value. “Driving pressure,” “respiratory failure,” and “titration” are the three keywords still maintaining a high brush value until now. The largest and smallest cluster of the co-cited references are “obstructive lung diseases” (#0, size: 97) and “lung perfusion” (#20, size: 5). Co-cited reference “Frerichs I, 2017, THORAX” (modularity change rate: 98.49) has the highest structural variability. Categories with most and least interdisciplinary crossing are “ENGINEERING” and “CRITICAL CARE MEDICINE.”

**Conclusions:**

EIT is a valuable technology for clinical lung monitoring, gradually converting from imaging techniques to the clinic. Research hot spots may continue monitoring techniques, the ventilation distribution of acute respiratory distress syndrome (ARDS), and respiratory therapy strategies. More diversified lung function monitoring studies, such as lung perfusion and interdisciplinary crossing, are potentially emerging research trends.

## Introduction

Electrical impedance tomography (EIT) is a non-invasive, radiation-free functional imaging technology invented over three decades ago, with the real-time application of monitoring global and regional lung function and ventilation distribution at the bedside (1). Recently, in the development of evidence-based medicine, a growing number of studies have confirmed that EIT is a useful tool in optimizing individual ventilator parameters, improving gas exchange, increasing oxygen levels, and decreasing ventilator-induced lung injury in respiratory failure (2–4). Clinical trials have reported that patients with acute respiratory distress syndrome (ARDS) could benefit from EIT-guided respiratory therapy (5, 6). With the clinical experience and the progress of clinical studies, topics regarding EIT clinical applications have been presented in different professional journals and scientific conferences. Much more attention has been given to the clinical applications of EIT. An intuitive overview and explicit research trends of clinical applications of EIT are beneficial for researchers to improve knowledge uptake, identify scientific advances, hot spots, research trends, and cooperative relationships as well as promote interdisciplinary cooperation. However, the studies that show the emerging trends and hot spots of publications in the field have not been reported.

A bibliometric analysis analyzes research publications based on big data and artificial intelligence (AI) (7). Research publications play an essential role in transmitting the process information of scientific development in a certain research field. AI has an innate advantage in dealing with huge amounts of data, e.g., enormous publications, and is more convenient in interpreting different quantitative rules in the network world. Therefore, a bibliometric analysis is used to evaluate contributions to a research field, including those by countries, institutions, authors, and journals (8). With the continuous improvement in the performance and effect of machine learning, AI has promoted the upgrading of bibliometric technology going deeper into investigation hot spots and research trends (9).

To explore the current status, emerging trends, and hot spots of EIT in clinical applications, a bibliometric analysis was conducted on the topic for the past two decades. This study helps researchers identify the most significant and impactful articles that highlight the characteristics of EIT application in clinical lung monitoring, and provide valuable insights into the most noteworthy research landscape, and forecast future work.

## Methods

### Data Sources and Search Strategies

The Web of Science (WoS) database, which has a rich collection of scientific literature, is commonly chosen for bibliometric analysis. In this study, all data were retrieved from the Web of Science Core Collection (WoSCC). The search strategy was TS = (“electrical impedance tomography”) AND (“lung”), Time window: January 1, 2001, to May 29, 2021, Publication type: “Article,” “Review,” and “Letter,” Language: English. All investigators collected the literature on May 29, 2021, to avoid database update bias.

### Data Collection

Two investigators (QSJ and LZ) independently extracted all data, including publications, author, title, abstract, keywords, source, language, citation. Publications less relevant to clinical applications are defined as studies and reviews based on image processing, algorithm, equipment assembly not limited to the electrode, and belt optimization or exploring the monitoring of other biological directions. The data were saved in a text and a UTF-8 format from the WoS core collection and saved for further software analysis.

### Bibliometric Analysis

#### WoS Core Database Output Analysis

The intrinsic functions of the WoS core database and Microsoft Excel (version Microsoft 365) were used to describe the features of the publications, including the total number of literature, annual, national, institutional, individual article counts, research field distributions, and top-cited literature.

### Network Analysis

Our data were imported into the bibliometric analysis software VOS (VOS viewer 1.6.16, Leiden University, Leiden, the Netherlands) or CiteSpace 5.7R5 (Chen Meichao, Drexel University) using a UTF-8 or a text format for network analysis. VOS finished top citations, coauthorship, co-occurrence analysis of authors, institutions, countries, keywords, and other factors. The numbers of documents and citations and the strength of the links were recorded and visually rendered with corresponding symbol color and line weight. The reference cluster analysis, citation bursts, and timeline view analysis, structural variation analysis (SVA), category co-occurrence analysis, etc., were finished using CiteSpace. Cluster type, size, strength timeline, and landmark literature were analyzed and visual rendering with corresponding image features (10). Keyword timeline view and keyword strongest citation burst analysis were conducted to show the topic's time scale and strength. The SVA was conducted to show landmark literature's contribution to this field. The category analysis was used to evaluate disciplinary cooperation.

## Results

### Bibliometric Analysis of Publication Output

**Figure 1:**
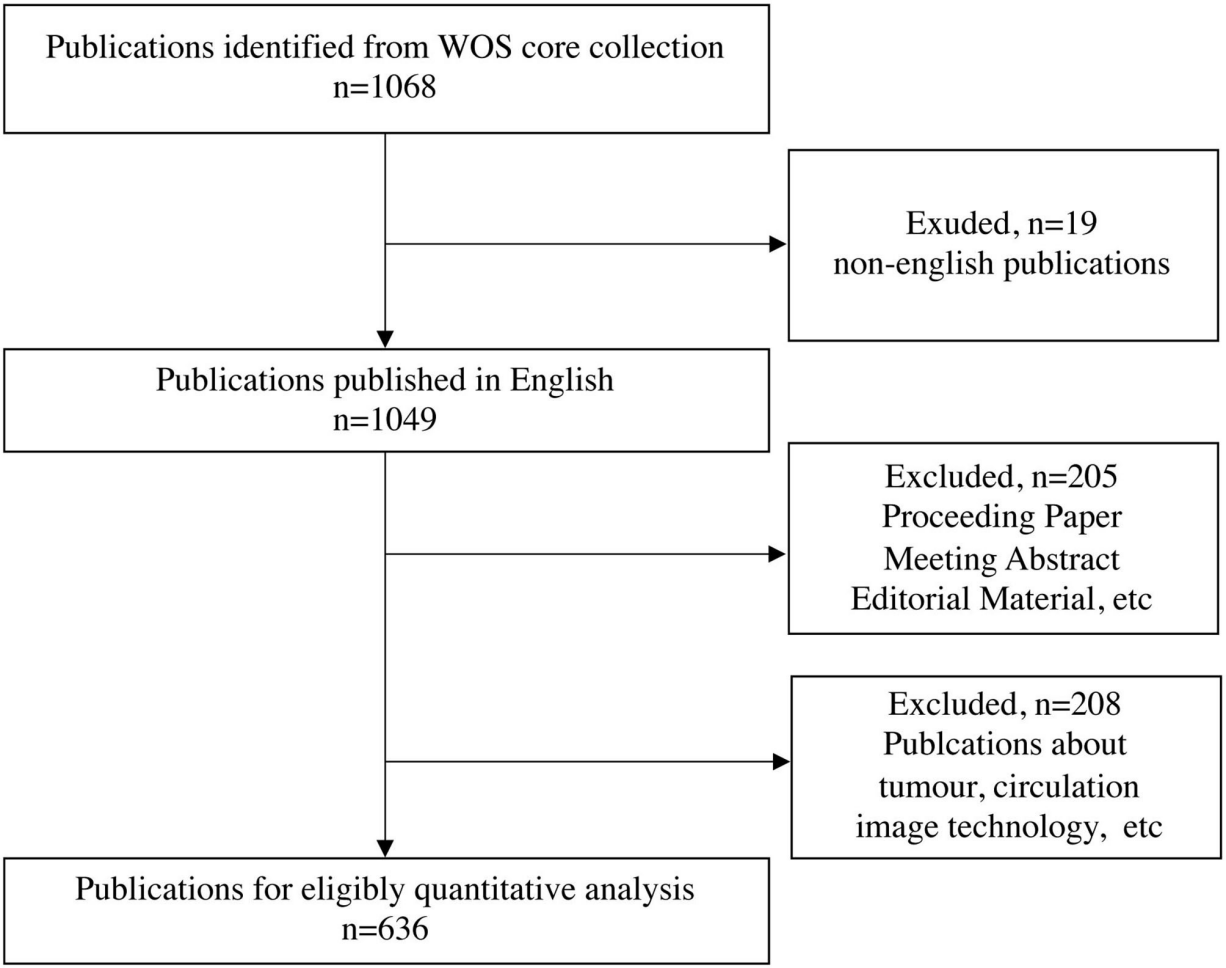
Flow chart of included publications. WoSC, Web of science center.

Following the retrieval strategy, 1,068 publications were identified and further screened. Finally, 636 research articles, reviews, and letters published in English on EIT applications in clinical lung monitoring were analyzed, and 432 publications were excluded (Figure 1).

### Growth Trend of Publications and Global Cooperation

**Figure 2:**
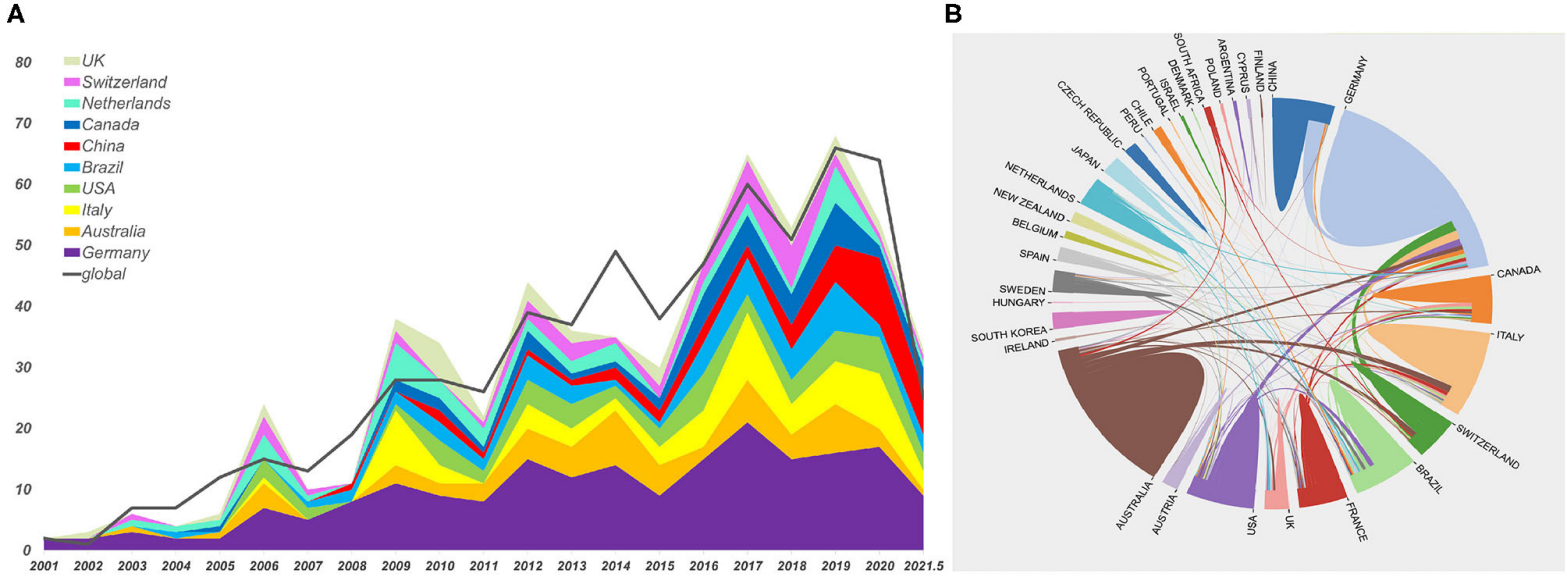
The number of annual publications, growth trends **(A)**, and country coauthorship analysis **(B)** of global and top 10 countries on electrical impedance tomography (EIT) applications in clinical lung monitoring research from 2001 to May 29, 2021. **(A)** The gray line indicated the trend of the annual world publication number. The color indicated different countries. The width of the color plate indicated the annual publication number of each country. **(B)** The color indicated countries; color plate size indicated the number of publications; the thickness of lines indicated linkage strength.

The literature counts between 2001 and 2020 illustrated the growth trend and global geographic distribution of publications in the field. Following the WoSCC database, 48 countries contributed to publications on EIT related to clinical lung monitoring. The global and top 10 countries in publications are indicated in Figure 2A. Overall, the annual EIT global publication number is increasing. Germany contributed the largest number of publications and has the most active cooperation on EIT applications in clinical lung monitoring studies with other countries (Figure 2B). Global national cooperation and countries collaborating most with Germany were indicated in Supplementary Figure 1.

### Analysis of Top Publications and Reference Articles

The ranking of the top 10 institutions, journals, authors of publications, and reference articles cited using EIT clinical lung monitoring publications is indicated in Table 1. Universities and their affiliated hospitals are the primary institution types contributed to publications in our research field. The department of University Medical Center Schleswig-Holstein (Univ Med Ctr Schleswig-Holstein) (publications: 84 and citing frequency: 2,205) owns most publications, Furtwangen University (Furtwangen Univ) (publications: 57 and citing frequency: 1,000) and University of São Paulo (Univ São Paulo) (publications: 49 and citing frequency: 2,061) ranked the second and third. Fourth Military Medical University (Fourth mil Med Univ) (publications: 30 and citing frequency: 134) ranked ninth and was the only Chinese institution to enter the top ten. Close cooperation can be observed in most productive and cited institutions (Supplementary Figure 1). The journal of Physiological Measurement (PHYSIOL MEAS) (105 publications, cited frequency 2,056), Critical Care (CRIT CARE) (33 publications, cited frequency 784), Intensive Care Medicine (INTENS CARE MED) (31 publications, cited frequency 1,723), Acta Anaesthesiologica Scandinavica (ACTA ANAESTH SCAND; 25 publications, cited frequency 360), and Critical Care Medicine (CRIT CARE MED; 23 publications, cited frequency 1,115) had the top five numbers of EIT publications in lung monitoring. Three of them are critical care-specialized journals. Inéz Frerichs (Frerichs, I; 116 publications, cited frequency 3,609) published most publications in the research field, followed by Zhao Zhanqi (Zhao ZQ; 43 publications, cited frequency 896), and Andy Adler (Adler A; 34 publications, cited frequency 1,113). Frerichs owned the largest author cooperative relationships as the most productive author. Furthermore, active cooperation could be found between high publishing and cited authors (Supplementary Figure 1). The top 10 most cited reference articles consisted of three reviews, four animal studies, and three clinical studies, “Victorino JA, 2004, AM J RESP CRIT CARE” (cited frequency 196), a clinical study, was a reference article with the most cited frequency (11).

**Table 1:** The top 10 institutions, journals, authors of publications, and reference articles on electrical impedance tomography (EIT) lung monitoring.

**Rank**	**Institution**	**Publications**	**Citations**	**Journal**	**Publications**	**Citations**	**Author**	**Publications**	**Citations**	**Cited reference**	**Citations**
1	Univ Med Ctr Schleswig Holstein	84	2,205	Physiol Meas	105	2,056	Frerichs, I	116	3,609	Victorino JA, 2004, AM J RESP CRIT CARE (11)^##^	196
2	Univ São Paulo	49	2,061	Intens Care Med	31	1,723	Zhao, ZQ	43	896	FrerichsI, 2017, THORAX (12)^*^	147
3	Univ Milan	37	803	Crit Care Med	23	1,115	Adler, A	34	1,113	Frerichs I, 2002, J APPL PHYSIOL (13)^#^	138
4	Furtwangn Univ	57	1,000	Crit Care	33	784	Leonhardt, S	28	814	Adler A, 2009, PHYSIOL MEAS (14)^*^	109
5	RWTH Aachen Univ	35	1,044	Curr Opin Crit Care	22	625	Tingay, D	27	628	Zhao ZQ, 2009, INTENS CARE MED (15)^##^	98
6	Royal Childrens Hospi	32	662	J Appl Physiol	18	586	Weiler, N	25	991	Meier T, 2008, INTENS CARE MED (16)^#^	95
7	Carleton Univ	38	1,152	Acta Anaesth Scand	25	360	Mauri, T	23	839	Frerichs I, 2000, Physiol Meas (17)^*^	94
8	Univ Melbourne	31	440	Resp Care	18	287	Amato, M	21	1,297	Hinz j, 2003, CHEST (18)^#^	92
9	Fourth mil Med Univ	30	134	PLoS ONE	17	162	Schibler, A	20	666	Brower RG, 2000, New Engl J Med (19)^##^	89
10	Murdoch Childrens Res Inst	29	602	Am J Resp Crit Care	16	1,128	Moeller, K	35	554	Frerichs I, 2006, Am J Resp Crit Care (20)^#^	86

*Cited reference: the references articles of publications on EIT applications in clinical lung monitoring involved in the analysis*.

* *Review*.

# *Animal research*.

## *Clinical research*.

### Bibliometric Analysis of Keyword Co-occurrence and Topic Trends

#### Keyword Co-occurrence Network and Overlay Analysis

**Figure 3:**
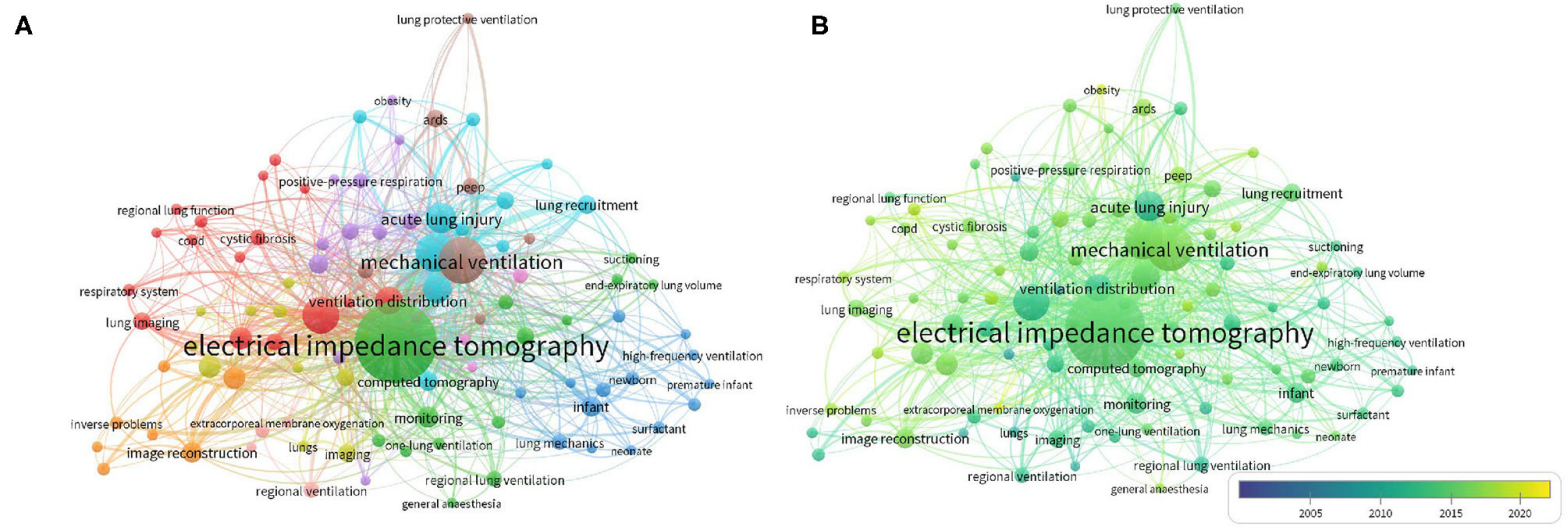
The keyword co-occurrence network and overlay analysis on EIT lung monitoring research from 2001 to May 29, 2021. **(A)** Network visualization of the keyword co-occurrence. **(B)** Overlay visualization of keyword co-occurrence. The color indicated various clusters **(A)** and the average publication year **(B)**. The circle size indicated the number of occurrences, and the thickness of lines indicated the linkage strength. The color indicated the average publication year of keywords. Circle size indicated the number of occurrences; the thickness of lines indicated the strength of the linkage. The distance between circles indicated their relationship.

One thousand two hundred keywords were identified from the included articles. A total of 100 keywords that occurred five or more times were defined as high-frequency keywords and enrolled in a co-occurrence network and overlay analysis. The keywords co-occurred in 10 clusters, 1,578 links, and 3,560 total link strengths (Figure 3A). “EIT” (occurrences, 304; total link strengths, 557) takes the top of highly frequency keyword, with a strong co-occurrence to “mechanical ventilation” (occurrences, 99; link strengths with EIT, 46), “acute respiratory distress syndrome” (occurrences, 67; link strengths with EIT, 40), “acute lung injury” (occurrences, 42; link strengths with EIT, 22), and “positive end-expiratory pressure (PEEP)” (occurrences, 38; link strengths with EIT, 19). The next top four high-frequency keywords were “mechanical ventilation” (occurrences, 99; total link strength, 259), “acute respiratory distress syndrome” (occurrences, 67; total link strength, 188), “PEEP” (occurrences, 38; total link strength, 116), and “ventilation distribution” (occurrences, 35; total link strength, 89). The top ten highly frequent keywords are ranked in Table 2.

**Table 2:** Top 10 highly frequent keywords from the included publications on EIT lung monitoring (*n* = 100).

**Rank**	**Keyword**	**Occurrences**	**Total link strengths**
1	Electrical impedance tomography	304	557
2	Mechanical ventilation	99	259
3	Acute respiratory distress syndrome	67	188
4	Positive end-expiratory pressure	38	116
5	Ventilation distribution	35	89
6	Image reconstruction	19	48
7	Infant	19	56
8	Lung recruitment	16	49
9	Ventilator-induced lung injury	14	39
10	Cystic fibrosis	12	22

The overlay analysis of the keywords representing the topic trends of EIT applications in clinical lung monitoring. The color distribution of keywords in the overlay visual map showed different periods (Figure 3B). The top 10 earliest and latest keywords are summarized in Table 3. “Noninvasive monitoring” (blue, occurrences 6, Avg, pub, year: 2008.17) was the earliest co-occurrence keyword, and “extracorporeal membrane oxygenation” (yellow, occurrences 5, Avg, pub, year: 2019.60) was the latest keyword (Supplementary Figure 2). “Ventilation distribution” (occurrences, 35; Avg, pub, year, 2012.03) had the highest co-occurrence frequency in the earliest keywords, and “PEEP” (occurrences, 13; Avg, pub, year, 2017.00) had the highest co-occurrence frequency in the latest keywords.

**Table 3:** The top 10 earliest and latest keywords of included publications on EIT lung monitoring (*n* = 100).

**Earliest**				**Latest**		
**Rank**	**Keyword**	**Occurrences**	**Avg, pub, year**	**Keyword**	**Occurrences**	**Avg, pub, year**
1	Non-invasive monitoring	6	2008.17	Extracorporeal membrane oxygenation	5	2019.60
2	Pulmonary edema	5	2008.60	Obesity	5	2019.00
3	Intensive care unit	5	2009.60	Lung perfusion	6	2017.67
4	Regional lung ventilation	11	2011.00	High-flow nasal cannula	6	2017.33
5	Premature infant	5	2011.40	Overdistention	6	2017.33
6	Diagnostic imaging	8	2011.75	Pulmonary function testing	5	2017.20
7	Regularization	6	2011.83	Transpulmonary pressure	6	2017.17
8	Regional ventilation	11	2011.91	peep	13	2017.00
9	Ventilation distribution	35	2012.03	Acute respiratory distress syndrome	5	2017.00
10	Lung impedance	7	2012.14	General anesthesia	5	2017.00

*Avg, pub, year: Average co-occurrence year (to the nearest 2 decimal place)*.

### Keyword Burst Value Analysis

**Figure 4:**
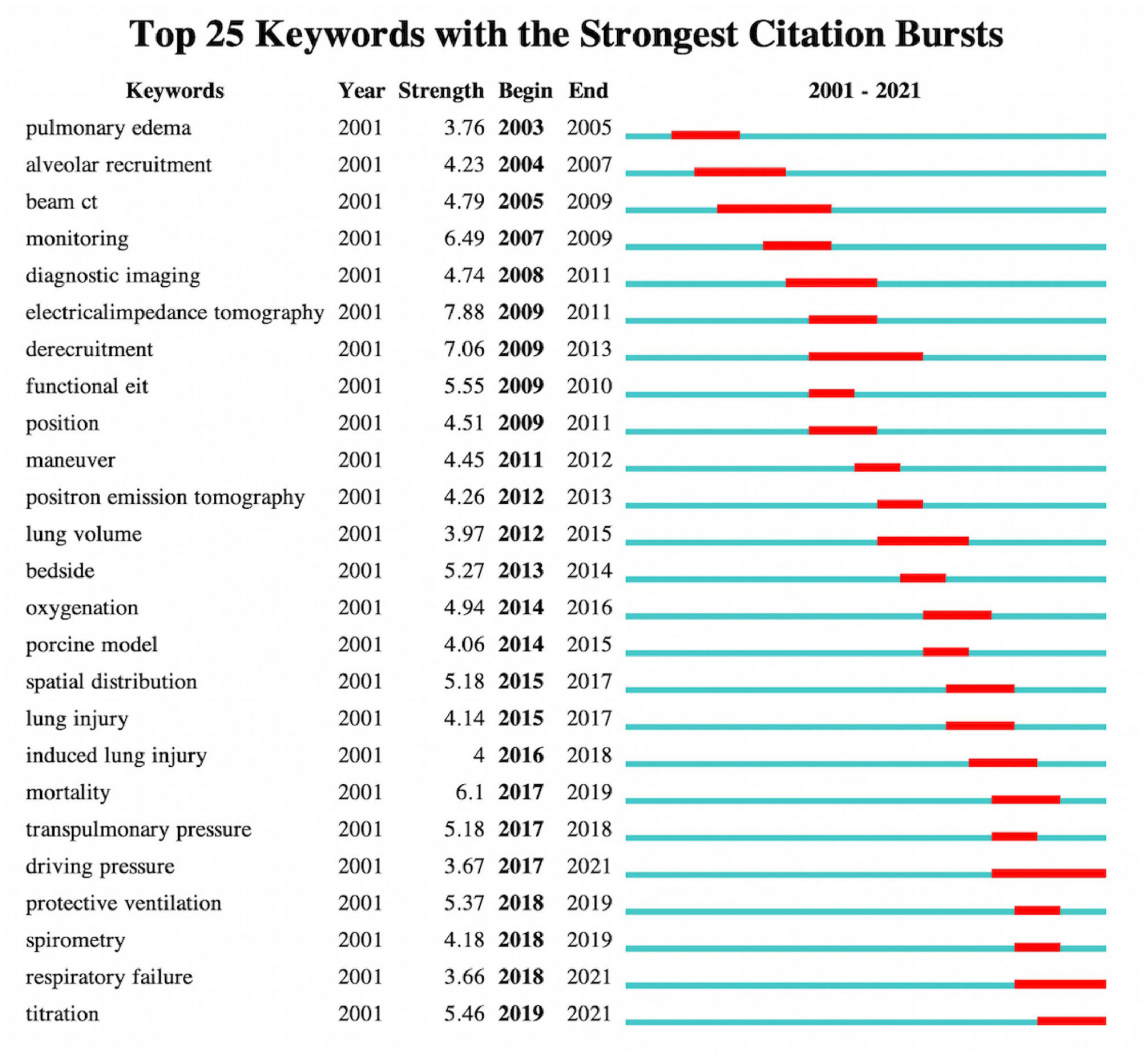
The top 25 keywords with the strongest burst value on EIT applications in clinical lung monitoring research from 2001 to May 29, 2021. The dark blue bar showed that the years in which keywords received slight increases in co-occurrence, the red bar indicates that co-occurrence rose sharply.

The keyword burst value analysis identified the hot spot keywords that have attracted the attention of peer investigators within a certain period. The top 25 keywords with the strongest burst value are summarized in Figure 4. During the entire period from 2003 to 2021, “electrical impedance tomography” (strength 7.88) had the highest burst strength, followed by “derecruitment” (strength 7.06), “monitoring” (strength 6.49), “mortality” (strength 6.1), and “functional EIT” (strength 5.55). Separately in 2001–2017, keywords about ventilation distribution monitoring device including “EIT,” “derecruitment,” “monitoring,” “bedside” (strength 5.27), and “spatial distribution” (strength 5.18) were strongly concerned, and in 2017–2021, keywords about ventilation injury and strategy, such as “protective ventilation” (strength 5.37), “titration” (strength 5.46), “transpulmonary pressure” (strength 5.18), “driving pressure” (strength 3.67), and induced “lung injury” (strength 4) were strongly cited. The keywords that still maintained a high brush value until now are “driving pressure,” “respiratory failure” (strength 3.66), and “titration” (strength 5.46), which may be the recent hot spot topics of investigators.

### Bibliometric Analysis of Co-cited Reference Analysis

#### Co-cited Reference Clustering and Time Evolution Analysis

**Figure 5:**
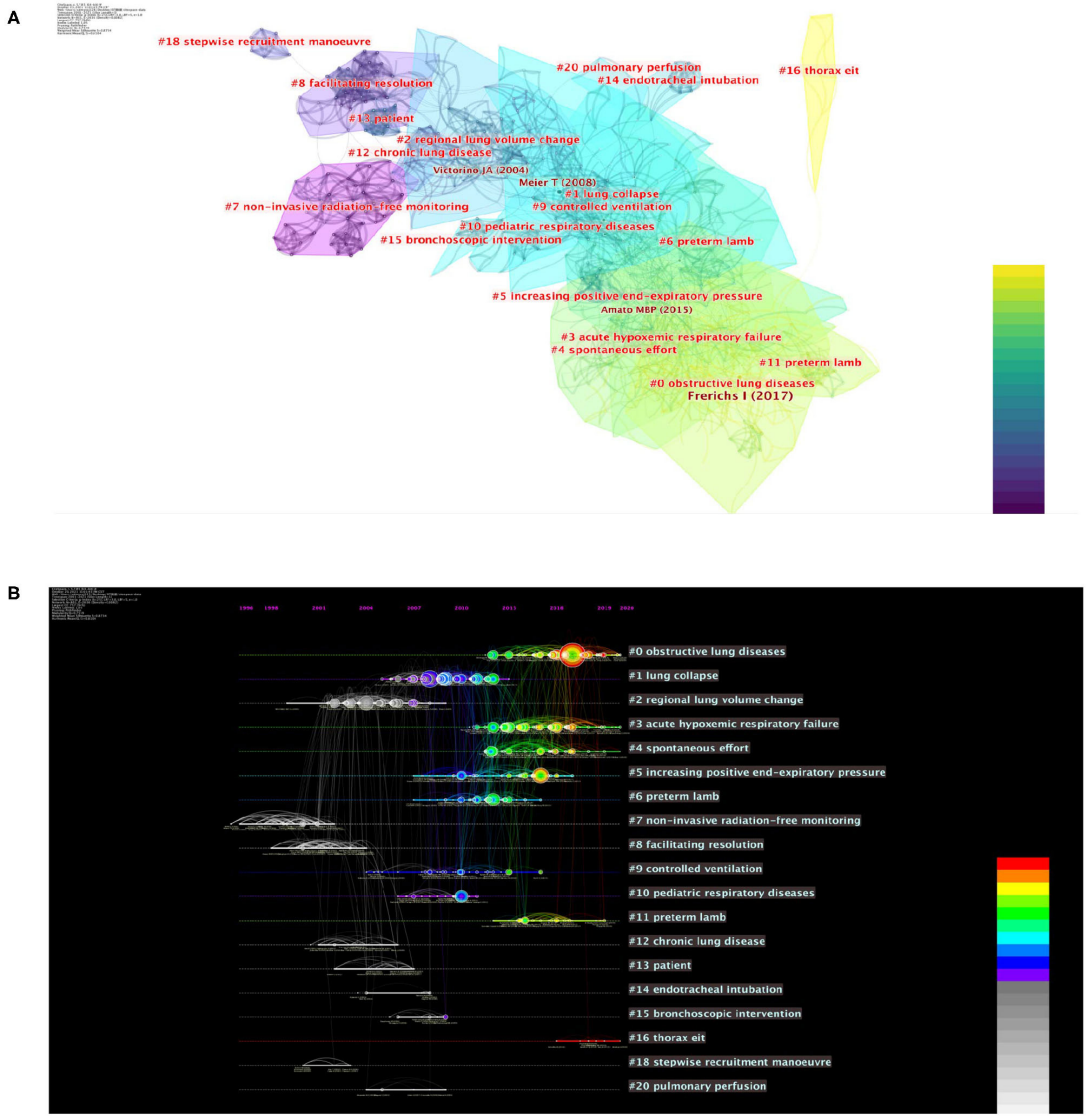
The co-cited reference clustering and time evolution analysis on EIT applications in clinical lung monitoring research from 2001 to May 29, 2021. **(A)** Co-cited reference network clustering analysis. The color indicates the publication year. The descending order vertically indicated cluster size. The line thickness indicated the strength of linkage. **(B)** The co-cited reference time evolution analysis. The color indicates the publication year. The colored curves represent co-citation links added in the year of the corresponding color. The large-sized nodes indicated they are either highly cited or have citation bursts or both. The descending order vertically indicated cluster size. The publication year is indicated above the map.

The co-cited reference clustering report exhibited a completely mean silhouette of 0.87 and a whole modularity Q score of 0.76, indicating that the clustering effect is efficient and convincing and the features and definition of every subdomain were distinct. Within the analysis, publications on EIT lung monitoring research were divided into 20 clusters (Figure 5), a vertically descending order showed cluster size, and cluster labels were obtained using the log-likelihood ratio (LLR) and mutual information (MI). The largest cluster is “obstructive lung diseases” (#0, size: 97) and the smallest cluster is “lung perfusion” (#20, size: 5). The next five largest clusters are “lung collapse” (#1, size: 94), “regional lung volume” (#2, size: 81), “acute hypoxemic respiratory failure” (#3, size: 72), “spontaneous effort” (#4, size: 66), and “increasing PEEP” (#5, size: 52). The large six clusters are summarized in Supplementary Table 1. Co-cited reference time evolution analysis shows that in the largest six clusters, “lung collapse” (1#), “regional lung volume change” (2#), and “increasing PEEP” (3#) were highly cited before 2010. “Obstructive lung disease” (0#), “regional lung volume change” (3#), and “acute hypoxemic respiratory failure” (4#) are highly cited since 2010 and kept as citation hotspot until now. Interestingly, despite the Q score, our result showed the two clusters (#6, #11) labeled as “Preterm lambs” using LLR.

### Co-cited Reference Burst Value and SVA

**Figure 6:**
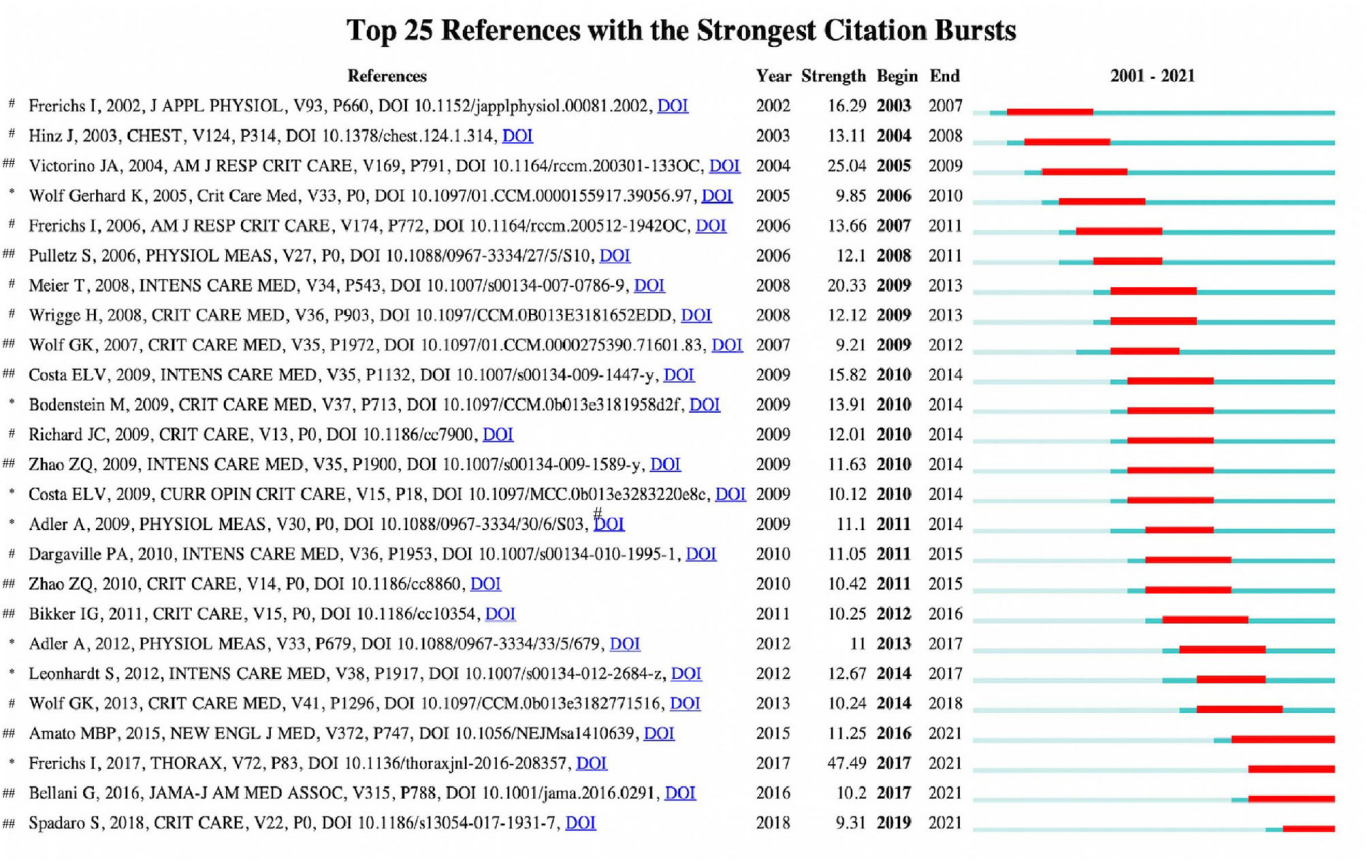
The top 25 cited references with the strongest burst value on EIT applications in clinical lung monitoring research from 2001 to May 29, 2021. The dark blue bar indicated the years in which keywords slightly increased as a co-occurrence. In contrast, the red bar shows that co-occurrence rises sharply. *Review, ^#^Animal research, and ^##^Clinical research.

The top 25 co-cited references with the strongest burst value on EIT applications in clinical lung monitoring from 2001 to 2020 are summarized in Figure 6. Top co-cited references are mostly from the specialty journals of respiratory and critical care, eight animal studies, seven reviews, and 10 clinical studies were included, and references began to burst since 2003, the publication “Frerichs I, 2017, THORAX” (12) (strength 47.45) had the highest burst strength, next four references with the burst strength above 15 were “Victorino JA, 2004, AM J RESP CRIT CARE” (11) (strength 25.04), “Meier T, 2008, INTENS CARE MED” (16) (strength 20.33), “Frerichs I, 2002, J APPL PHYSIOL” (13) (strength 16.29), and “Costa ELV, 2009, INTENS CARE MED” (21) (strength 15.82). The average duration of the burst value lasts for 3–4 years, “Amato MBP, 2015. NEW ENGL J MED” (22) (strength 11.25, 2016–2021) is the only reference with a burst duration of more than 5 years and is still highly cited today with the other three literature “Frerichs I, 2017, THORAX” (12) (strength 47.45, 2017–2021), “Bellani G, 2016, JAMA-J AM MED ASSOC” (strength 10.2, 2017–2021) (23), and “Spadaro S, 2018, CRIT CARE” (24) (strength 9.31, 2019–2021). Co-cited reference burst value analysis showed the publications that are highly frequently cited in a specific period imply rising research interests of the topic of these publications.

To identify the capacity of cited references to make extraordinary or unexpected connections across distinct clusters and detect the potential landmark studies in the EIT research field, the SVA was conducted. The top five structurally variational references are listed in Table 4. The clustering and clustering time evolution situations of these articles are marked in Figure 7. The publication with the highest modularity change rate in our data set is “Frerichs I, 2017, THORAX” (12) (the modularity change rate: 98.49; cited frequency: 147), spanning three clusters: “obstructive lung diseases” (#1), “acute hypoxemic respiratory failure” (#3), and “spontaneous effort” (#4). “Leonhardt S, 2012, INTENS CARE MED” (26) (the modularity change rate: 95.06; cited frequency: 96); “Adler A, 2012, PHYSIOL MEAS” (25) (the modularity change rate: 93.82; cited frequency: 108) spanning the same clusters “pediatric respiratory diseases” (#10) and #1 “lung collapse” (#1) took the second and third place. Three of the top five structural variational references were review articles, among them “Leonhardt S, 2012, INTENS CARE MED” (26), a review summary of the state-of-the-art in EIT for ventilation and perfusion imaging, had the lowest number of citations, but took the second place of the modularity change rate. All the top five structural variation references have a burst value of at least 11. Boundary-spanning ideas may contribute to interdisciplinary scientific and technological progress and raise significant concerns among investigators.

**Table 4:** The top five co-cited references with the strongest structural variation value.

**Title**	**Publication type**	**Author**	**Publication year**	**Journal**	**Modularity change rate**	**Citations**	**Cluster linkage**	**Centrality divergence**
Chest electrical impedance tomography examination data analysis terminology clinical use and recommendations: consensus statement of the TRanslational EIT developmeNt stuDy group^*^	Review	Frerichs I	2017	Thorax	98.49	147	0.73	0.16
Electrical impedance tomography: the holy grail of ventilation and perfusion monitoring?^*^	Review	Leonhardt S	2012	Intens Care Med	95.06	96	0.03	0.82
Whither lung EIT: Where are we where do we want to go and what do we need to get there?^*^	Review	Adler A	2012	Physiol Meas	93.82	108	3.75	0.59
Detection of local lung air content by electrical impedance tomography compared with electron beam CT^#^;	Original Article	Frerichs I	2002	J Appl Physiol	90.68	138	67.04	0.35
Imbalances in regional lung ventilation—A validation study on electrical impedance tomography^##^	Original Article	Victorino JA	2004	Am J Resp Crit Care	88.57	196	24.94	0.37

*Modularity change rate: the structural changes of the underlying network are induced due to connections with a contribution from new publications. Cluster linkage: an effect of between-cluster links before and after a new paper becomes available. Centrality divergence: the structural variations arising from a new article based on the divergence of the distributions between the central measures of all nodes in the network before and after the information from the new article are considered*.

* *Review*,

# *Animal research*,

## *Clinical research*.

**Figure 7:**
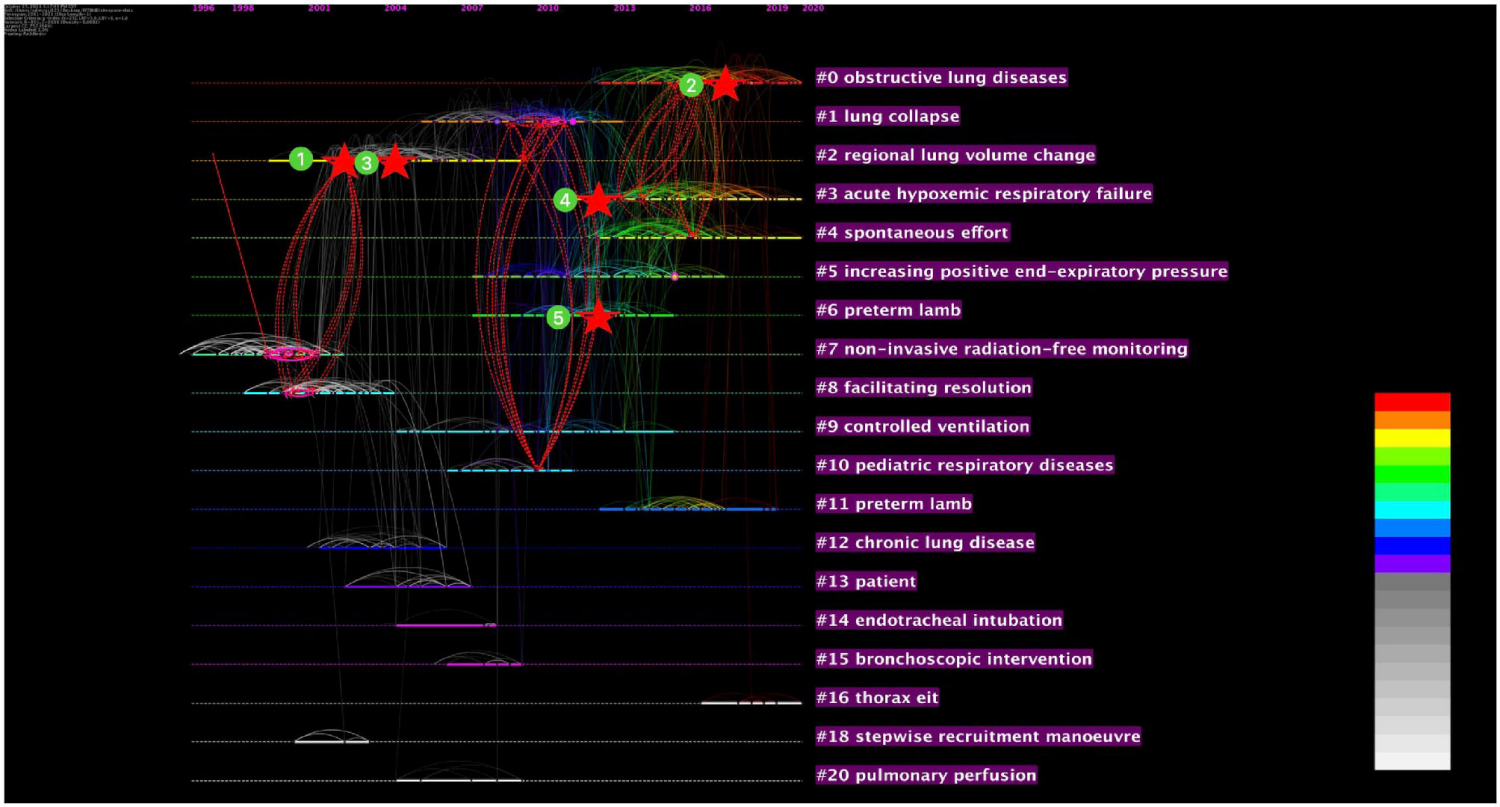
The co-cited reference structural variation analysis (SVA) on EIT lung monitoring research from 2001 to May 29, 2021 (Modularity *Q* = 0.76; Weighted Mean Silhouette *S* = 0.87). The stars indicate citing articles, and the dashed lines indicate novel co-citation links. [①: Victorino et al. (11); ②: Frerichs et al. (12); ③: Frerichs et al. (13); ④: Adler et al. (25); ⑤: Leonhardt et al. (26)].

### Category Co-occurrence Analysis

**Figure 8:**
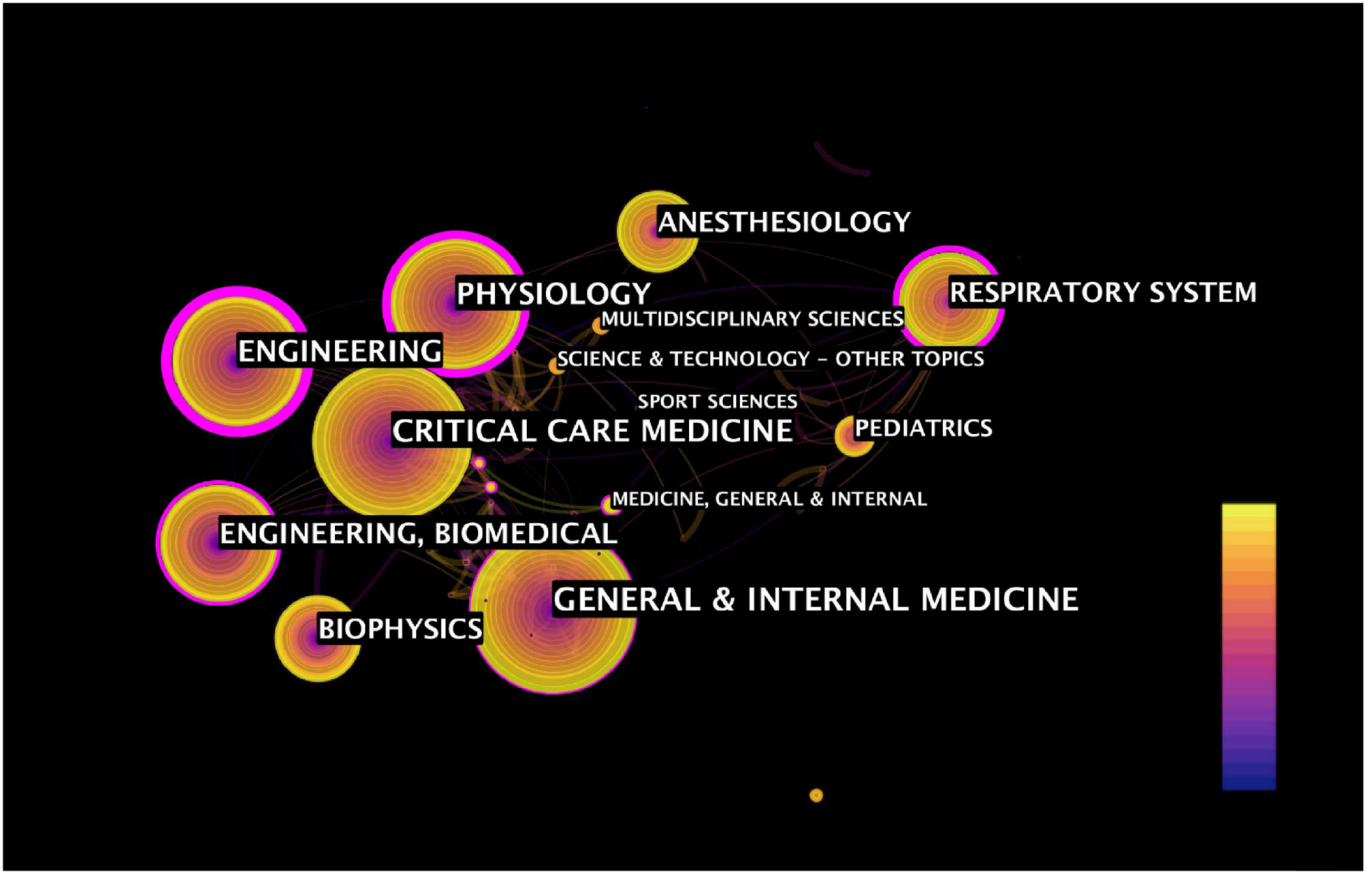
The category co-occurrence analysis on EIT applications in clinical lung monitoring research from 2001 to May 29, 2021. The circle indicates the category; the circle size indicates publication number, the color shows the first publication year. The thickness of the lines indicated the strength of the linkage. The purple ring indicates centrality above 0.1 of categories. Centrality: metric score indicates the catenation value of the category in the entire network structure, ranges from 0–1, above 0.1 indicate turning points.

The category co-occurrence map showed 62 disciplines cross and penetrate each other with 172 link lines (Figure 8). The largest five categories are “GENERAL & INTERNAL MEDICINE” (frequency: 213; Centrality: 0.24), “CRITICAL CARE MEDICINE” (frequency: 192; Centrality: 0.01), “PHYSIOLOGY” (frequency: 136; Centrality: 0.48), “ENGINEERING” (frequency: 136; Centrality: 0.84), and “ENGINEERING, BIOMEDICAL” (frequency: 125; Centrality: 0.24). Among the major categories, “ENGINEERING” with a high centrality of 0.84 showed currently active interdisciplinary crossing, “CRITICAL CARE MEDICINE” with a centrality score of 0.01 represents weak interdisciplinary crossing.

## Discussion

This study performs a bibliometric analysis of publications on EIT applications in clinical lung monitoring from 2001 to 2021. A total of 636 publications were analyzed (Figure 1), the result reflects the development process, the current situation, emerging trends, and hot spots of the research field over time. The number of publications showed an approximately increasing trend from 2001 to 2020, suggesting that EIT has become a concerned research field (Figure 2).

The publication composition indicated that Germany as the pioneer country published the largest number of articles on EIT with lung monitoring every year from 2001 to 2021 (Figure 2). It was noticed that there was a remarkable increase in Chinese publications after 2019, this may be because of the availability of EIT equipment for clinical research after obtaining the license from the China Food and Drug Administration in 2014 (1) and increasing demand for bedside non-invasive lung monitoring technology after the outbreak of COVID-19 (27–29).

Citation counts of institutions, journals, authors, and reference articles generally represent scientific acknowledgment for peer investigators (30). Highly cited institutions, authors, and countries cooperated closely and published a considerable number of publications, as shown in Table 1 and Supplementary Figure 1. International scientific cooperation can enhance research quality and promote medical progress (31), follow the trend of international diversification, join online or offline international specialized conferences, and seek interdisciplinary potential and effective ways to promote transnational cooperation. Journal citation analysis can provide reliable references for researchers to search literature or submit manuscripts (32). The journal Physiological Measurement has a tradition of collaborating with the EIT annual conference and publishes a special issue each year after the conference. It produced the highest number of publications and had the highest citation. Critical care-specialized journals contribute most publications and cited frequency in the research field as the most promising application direction of lung monitoring with EIT occurs in the ICU. The validation of animal or clinical studies between EIT and gold standards, such as CTs under physiological conditions or disease models (11, 13, 20), studies defining new parameter indicators from EIT (15, 16), and systematic reviews of EIT imaging technology and clinical applications with increasing clinical evidence (12) were highly cited (Table 1). All these results indicate that EIT, as a convenient lung function measuring device, plays an essential role in the field of daily and scientific research use in critical care and intensive care medicine.

Refining the research topic with keywords, in the last two decades, the primary research direction of EIT with clinical lung monitoring has focused on diagnostic imaging for ARDS, which is the most life-threatening and difficult respiratory syndrome worldwide and is possible to remain as research hot spots over the next few years. The biggest advantage of EIT technology is the potential to monitor the regional pulmonary function of intensive care patients at the bedside, and early studies focused on clinical use scenarios related to EIT, such as “noninvasive monitoring,” “intensive care unit,” and monitoring indicators that can be completed, such as “lung impedance” and “ventilation distribution.” With the clinical acceptance and promotion of the technology, clinical research of EIT more specifically focuses on optimal respiratory treatment strategies for ARDS, such as monitoring “regional mechanical ventilation distribution” and “PEEP titration” (Figure 4, Tables 2, 3), the controversies of protective ventilation in ARDS provided researchers the opportunities to study ARDS using EIT. With more recent experience in clinical use, the topics extend beyond ARDS back to EIT technology as exploring the ventilation statues of new respiratory treatment methods for acute respiratory failure (ARF), e.g., “high-flow nasal cannula” and “extracorporeal membrane oxygenation” as well as a specific population, e.g., “obesity” or subjects under “general anesthesia” become an emerging trend of EIT-related clinical studies (Table 3, Supplementary Figure 2).

Our keyword analysis also showed the understanding of EIT clinic applications on one topic changing over time, for example, “ARDS,” early burst keywords focus on the clinical phenomenon, such as “pulmonary edema.” With advances in clinical practice and scientific research, investigators found that the nature behind these phenomena was “lung injury” and subsequent research gradually focused on clinical intervention strategies, such as “lung protective ventilation.” The same holds true for the understanding of “lung recruitment”: early understanding may lie in some diseases causing pathophysiological changes as “alveolar collapse” and with the clinical introduction of EIT, it was recognized that such physiological changes can be visualized through diagnostic images as “lung recruitment” and “derecruitment” after which we can apply this topic to clinical treatments, such as “PEEP titration” (Figure 4). The depth and breadth of the clinical application of EIT have also changed over time. As mentioned earlier, the current clinical research of EIT is not limited to “ARDS” but an evolution to more generalized “ARF,” the Research Topic expands beyond the ventilation, such as “perfusion” and the clinical application scenario is not limited to the intensive care unit but anesthesia. These changes interact with the discipline's cognitive progress of disease and the clinical scientific research application of visual EIT monitoring.

References are essential for the selection, execution, and summary of scientific research. The high-quality literature of a specific research object can give a reference for this research direction and form a system through positive research accumulation. References of publications on EIT applications in clinical lung monitoring cluster toward the evaluation of ARDS lung ventilation like lung collapse and increasing PEEP in the early years (Figure 5). Presently, similar to keyword analysis, respiratory failure, regional ventilation, and extensive pulmonary function monitoring may become research hot spots in EIT clinical use. From our results, for topics with a high clustering intensity at an early age but not in recent years, e.g., “lung perfusion,” technical bottleneck, and clinical adaptation might be the reasons for changes.

*Focus on the content of cited reference*: Among studies with high burst values, in early days, animal studies that validated EIT and imaging gold standards (13, 18), and animal model studies (16, 20) had high burst values, as medium animals, such as pigs and sheep, are preferred for simulating respiratory physiology and establishing disease model for validation studies. This is vital for introducing EIT as a new technology for clinical ventilation monitoring. Since EIT was used in complex clinical scenarios, volunteer and patient studies (11, 21) are frequently cited. In addition, clinical studies introduced new parameters calculated by EIT (15, 33) and related articles in ARDS treatment (34) are also highly cited (Figure 6). Another highly cited type is reviews. The topic of EIT-related review started on the principles and EIT indications, and then focused on the summary of clinical applications, the exploration of new fields, “Frerichs I, 2017, THORAX” (12), a consensus statement of the translational EIT development study group provides examination, data analysis, terminology, and clinical use recommendations of EIT, had the highest burst strength three times higher than others. The literature “Amato MBP, 2015, NEW ENGL J MED” (22), an observational trial regarding driving pressure in ARDS without mentioning EIT, is the only reference with a burst duration of more than 5 years, and is still highly cited today (Figure 6). It was supposed that this article brings the investigators to the physiological changes of local ventilation during ARDS, which is exactly what EIT monitoring technology can approve. Our analysis suggests that a high-quality review or guideline from pioneers in the professional field may provide reliable evidence for the beginning of new studies on EIT related to clinical lung monitoring. Additionally, the progress and concept of clinical diseases, implying rising research interest trends, were also essential for promoting EIT's clinical use. As a visualized ventilation monitoring tool, a gradual understanding of EIT and its application need multiple levels of validation. with a deeper understanding of disease and EIT technology, the breadth of future researches will be expanded.

The structural variation analysis was conducted to find the co-cited reference spanning cluster boundaries. High structural variation references are high burst value reviews (Figure 7 and Table 4). “Frerichs I, 2017, THORAX (12),” a clinical application consensus gives a detailed clinical application protocol of EIT, spanning three clusters. “Leonhardt S, 2012, INTENS CARE MED (26)” reviewed EIT applications in ventilation and perfusion imaging and got a high modularity change rate relative to cited frequency. That is probably because it elaborated special respiratory monitoring using EIT, which gives an impetus to cross-category research. It is not difficult to infer that macro-overview or review depth in branch direction have a high reference value for research on EIT monitoring.

Some studies on EIT applications in clinical lung monitoring have the characteristics of interdisciplinary crossing. “PHYSIOLOGY,” “ENGINEERING,” and “RESPIRATORY SYSTEM,” the three categories, represent the technical foundation and operational value of EIT, which confirmed the active interdisciplinary crossing. “CRITICAL CARE MEDICINE” is a hot subject, but building a collaboration with other disciplines needs to be addressed in further research (Figure 8).

## Strengths and Limitations

This study is the first bibliometric analysis evaluating publications on the application of EIT in clinical lung monitoring extracted from the WoS core database. This study provides a quick and objective reference for interested researchers by visualizing the current status, hot spots, and emerging trends of EIT from 2001 to 2021. However, some limitations are inevitable. First, the WoSCC database is updated continuously and dynamically. There might be some new data missing, even all the database searches were conducted in 1 day. Second, to obtain more subject-oriented research, only English original articles and reviews about clinical applications were included, the sample size of articles finally included in the analysis is limited and a discrepancy may exist between our results and the real publication characteristics. Finally, the multiple expressions of author, institution, and keywords result in the dispersion of counts and clusters. Although these problems were addressed with the merge and normalization function of the software, they cannot be avoided completely.

## Conclusion

Electrical impedance tomography applications in clinical lung monitoring are concerned research fields from 2001 to 2021. Germany was a pioneer country in this research field, while the Univ Med Ctr Schleswig and Frerichs I achieved significant research results and contributed to the development of EIT research. Professional macro and in-depth review and interdisciplinary literature could give a reliable reference for EIT research, while cooperation would promote the development of the research field. Ventilation distribution in ARDS and respiratory therapy strategies were research focus in the past two decades and will continue as research hot spots. More diversified lung function monitoring techniques, such as lung perfusion and interdisciplinary crossing with EIT, are potential emerging research trends on EIT applications in clinical lung monitoring.

## Data Availability Statement

Publicly available datasets were analyzed in this study. This data can be found here: WoS database, http://isiknowledge.com/.

## Author Contributions

ZL, SQ, and ZZ have designed the study and drafted the manuscript. YY and SM have performed collected the data. ZL, SQ, and CC have analyzed the data. WL and YG have revised the manuscript. All authors have approved the final version.

## Funding

This work was supported by the Shanghai Jiao Tong University (No. YG2019ZDB04), Natural Science Research Project of Minhang District (No. 2019MHZ017), and Shanghai Renji Hospital Clinical Research and Cultivation Fund (No. PY2018-IIA-01).

## Conflict of Interest

ZZ receives a consulting fee from Dräger Medical. The remaining authors declare that the research was conducted in the absence of any commercial or financial relationships that could be construed as a potential conflict of interest.

## Publisher's Note

All claims expressed in this article are solely those of the authors and do not necessarily represent those of their affiliated organizations, or those of the publisher, the editors and the reviewers. Any product that may be evaluated in this article, or claim that may be made by its manufacturer, is not guaranteed or endorsed by the publisher.
