# Supplementary material for: Emerging Trends and Hot Spots of Electrical Impedance Tomography Applications in Clinical Lung Monitoring
Source: Front Med (Lausanne). 2022 Jan 31;8:813640. doi: 10.3389/fmed.2021.813640 (PMC8841839; doi:10.3389/fmed.2021.813640)
Supplement: Supplementary file 1 [file Data_Sheet_1.docx]

**Supplementary materials**

**Supplemental Fig. 1** The co-authorship analysis on EIT lung monitoring research from 2001 to 2021.5.29.

**Supplemental Fig. 2** The overlay visualization of the earliest and latest keywords co-occurrence on EIT applications in clinical lung monitoring research from 2001 to 2021.5.29.

**Supplement. Table1** The top 6 largest co-cited references clusters

**Supplement. Table2** Summary of publications mentioned in the manuscript

**
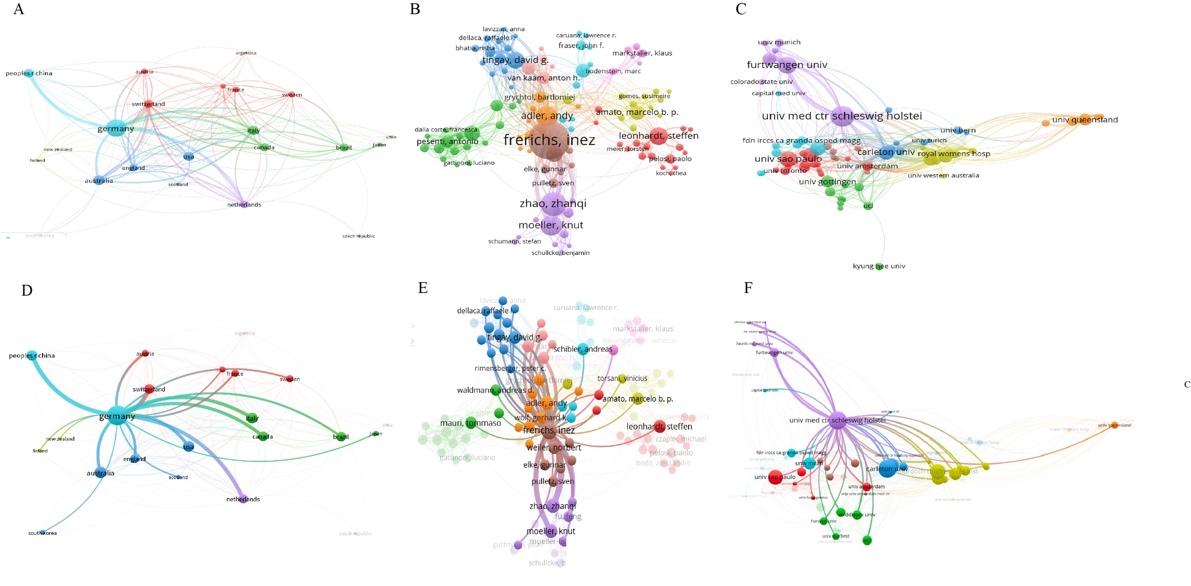
**

**Supplemental Fig. 1** The co-authorship analysis on EIT lung monitoring research from 2001 to 2021.5.29. The highest authors, institutions, and countries co-authorship with Frerichs I, University Medical Center Schleswig-Holstein and Germany. In the global cooperation of countries (A), authors (B) and institutions (C), the color indicates clusters, circle size indicates the number of publications, and the thickness of lines indicates the strength of linkage (A, B, and C). Country co-authorship with Germany is denoted as D, author co-authorship with Frerichs I is denoted as E, and institution co-authorship with University Medical Center Schleswig-Holstein is denoted as F. The color indicates countries (D), authors (E) and institutions (F), circle size indicates the number of publications, and the thickness of lines indicates the strength of linkage (D, E, and F).


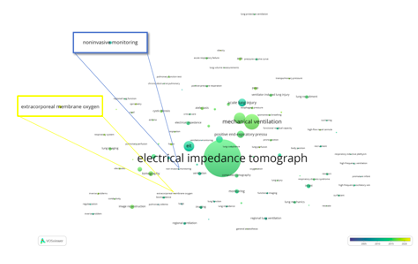


**Supplemental Fig. 2** The overlay visualization of the earliest and latest keywords co-occurrence on EIT applications in clinical lung monitoring research from 2001 to 2021.5.29. The color indicated the average publication year, circle size showed the number of occurrences, the distance between circles showed their relationship. The blue box indicated the earliest keyword. The yellow box indicated the latest keyword.

**Supplement. Table1 The top 6 largest co-cited references clusters**

| Cluster ID | Size | Silhouette | Label (LLR) | Label  (MI) | Mean  (Citee Year) | modularity  Q score |
| --- | --- | --- | --- | --- | --- | --- |
| 0 | 97 | 0.846 | obstructive lung diseases  (179.4, 1.0E-4) | open-lung approach (1.86); regional opening (1.86); clinical usefulness (1.86); occult pendelluft (1.86); thorax eit (1.86); pig model (1.86); bedside monitoring (1.86); tissue properties (1.86); gravitational distribution (1.86); monitoring pulmonary perfusion (1.86); ventilation condition (1.86); lung abnormalities (1.86); surfactant treatment (1.86); perioperative assessment (1.86); paediatrics-a survey (1.86); clinical diagnosis (1.86); semi-siamese u-net (1.86); using lung density value (1.86); pulmonary perfusion (1.86); end-expiratory lung volume change (1.86); changing body position (1.86); patient (1.86); pleural effusion drainage (1.68); facilitating resolution (1.68); intratidal compliance profile analysis (1.68); breathing neonate (1.68); study protocol (1.68); lung function test (1.68); open abdominal surgery (1.68); individualized peep titration (1.68); of-bed elevation (1.68); bronchoscopic intervention (1.68); gravity-dependent lung ventilation (1.68); acute injury (1.68); protective mechanical ventilation (1.68); ventilation homogeneity (1.68); whither lung eit (1.68); neonatal piglet model (1.68); different body position (1.68); different bedside technique (1.68); lung recruitment maneuver (1.68); conventional lung (1.68); endotracheal tube position (1.68); interstitial lung diseases (1.68); regional ventilation inhomogeneity (1.68); clinical scenario (1.57); paediatric intensive care (1.57); endotracheal intubation (1.57); lung-function test (1.57); blunt chest trauma (1.57); unilateral empyema (1.57); varying level (1.57); ventilation scintigraphy (1.57); tidal breathing (1.57); regional filling characteristics (1.57); respiratory control (1.57); long-term porcine model (1.57); pressure support (1.57); severe multiple trauma (1.57); regional compliance (1.57); long-term ventilator-induced lung injury ventilation (1.57); randomized trial (1.5); severe ard (1.5); individual level (1.5); chest examination (1.5); lung recruitability (1.5); absolute lung resistivity (1.5); early postoperative ventilation distribution (1.5); visual anatomical lung ct (1.5); neonatal lung (1.5); arm position (1.5); alveolar ventilation (1.5); preterm lung (1.5); oscillatory volume (1.5); noninvasive high-frequency ventilation (1.5); following transition (1.44); noninvasive respiratory support (1.44); electron beam ct (1.44); preterm rabbit (1.44); overall ventilation inhomogeneities (1.44); different approaches (1.44); lung tissue properties (1.44); chronic lung disease (1.44); local lung air content (1.44); term-born infant (1.44); quantifying ventilation distribution (1.44); breathing lamb (1.44); bests positive end-expiratory pressure (1.44); supraglottic atomization (1.44); decremental positive end-expiratory pressure trial (1.44); receiving continuous positive airway pressure (1.44); histamine-provoked change (1.39); gravity effect (1.39); virus infection (1.39); ongoing pathophysiological (1.39); mechanical function (1.39); birth-weight infant (1.39); therapeutic development (1.39); gradual tidal inflation (1.39); assessing respiratory system (1.39) | 2016 | 0.76 |
| 1 | 94 | 0.82 | lung collapse (211.09, 1.0E-4) | regional opening (1.17); open-lung approach (1.17); clinical usefulness (1.17); occult pendelluft (1.17); thorax eit (1.17); pig model (1.17); bedside monitoring (1.17); tissue properties (1.17); gravitational distribution (1.17); monitoring pulmonary perfusion (1.17); ventilation condition (1.17); lung abnormalities (1.17); surfactant treatment (1.17); perioperative assessment (1.17); paediatrics-a survey (1.17); clinical diagnosis (1.17); semi-siamese u-net (1.17); using lung density value (1.17); pulmonary perfusion (1.17); end-expiratory lung volume change (1.17); changing body position (1.17); patient (1.17); of-bed elevation (1.05); ventilation homogeneity (1.05); whither lung eit (1.05); facilitating resolution (1.05); intratidal compliance profile analysis (1.05); breathing neonate (1.05); study protocol (1.05); lung function test (1.05); open abdominal surgery (1.05); individualized peep titration (1.05); bronchoscopic intervention (1.05); gravity-dependent lung ventilation (1.05); acute injury (1.05); pleural effusion drainage (1.05); protective mechanical ventilation (1.05); neonatal piglet model (1.05); different body position (1.05); different bedside technique (1.05); lung recruitment maneuver (1.05); conventional lung (1.05); endotracheal tube position (1.05); interstitial lung diseases (1.05); regional ventilation inhomogeneity (1.05); varying level (0.98); pressure support (0.98); endotracheal intubation (0.98); lung-function test (0.98); blunt chest trauma (0.98); unilateral empyema (0.98); clinical scenario (0.98); long-term ventilator-induced lung injury ventilation (0.98); ventilation scintigraphy (0.98); tidal breathing (0.98); respiratory control (0.98); long-term porcine model (0.98); severe multiple trauma (0.98); regional compliance (0.98); paediatric intensive care (0.98); regional filling characteristics (0.98); early postoperative ventilation distribution (0.93); alveolar ventilation (0.93); individual level (0.93); chest examination (0.93); randomized trial (0.93); preterm lung (0.93); oscillatory volume (0.93); lung recruitability (0.93); absolute lung resistivity (0.93); visual anatomical lung ct (0.93); severe ard (0.93); neonatal lung (0.93); noninvasive high-frequency ventilation (0.93); arm position (0.93); different approaches (0.89); lung tissue properties (0.89); quantifying ventilation distribution (0.89); following transition (0.89); noninvasive respiratory support (0.89); electron beam ct (0.89); breathing lamb (0.89); chronic lung disease (0.89); local lung air content (0.89); supraglottic atomization (0.89); receiving continuous positive airway pressure (0.89); preterm rabbit (0.89); overall ventilation inhomogeneities (0.89); bests positive end-expiratory pressure (0.89); term-born infant (0.89); decremental positive end-expiratory pressure trial (0.89); birth-weight infant (0.86); air distribution (0.86); gravity effect (0.86); mechanical function (0.86); histamine-provoked change (0.86); gradual tidal inflation (0.86); assessing respiratory system (0.86); individualized lung recruitment strategies (0.86); parabolic flight (0.86) | 2009 |  |
| 2 | 81 | 0.835 | regional lung volume change (189.96, 1.0E-4) | regional opening (0.44); open-lung approach (0.44); clinical usefulness (0.44); occult pendelluft (0.44); thorax eit (0.44); pig model (0.44); bedside monitoring (0.44); tissue properties (0.44); gravitational distribution (0.44); monitoring pulmonary perfusion (0.44); ventilation condition (0.44); lung abnormalities (0.44); surfactant treatment (0.44); perioperative assessment (0.44); paediatrics-a survey (0.44); clinical diagnosis (0.44); semi-siamese u-net (0.44); using lung density value (0.44); pulmonary perfusion (0.44); end-expiratory lung volume change (0.44); changing body position (0.44); patient (0.44); lung function test (0.39); ventilation homogeneity (0.39); regional ventilation inhomogeneity (0.39); facilitating resolution (0.39); intratidal compliance profile analysis (0.39); breathing neonate (0.39); study protocol (0.39); open abdominal surgery (0.39); individualized peep titration (0.39); of-bed elevation (0.39); bronchoscopic intervention (0.39); gravity-dependent lung ventilation (0.39); acute injury (0.39); pleural effusion drainage (0.39); protective mechanical ventilation (0.39); whither lung eit (0.39); neonatal piglet model (0.39); different body position (0.39); different bedside technique (0.39); lung recruitment maneuver (0.39); conventional lung (0.39); endotracheal tube position (0.39); interstitial lung diseases (0.39); regional filling characteristics (0.36); lung-function test (0.36); tidal breathing (0.36); respiratory control (0.36); endotracheal intubation (0.36); blunt chest trauma (0.36); unilateral empyema (0.36); varying level (0.36); clinical scenario (0.36); long-term ventilator-induced lung injury ventilation (0.36); ventilation scintigraphy (0.36); long-term porcine model (0.36); pressure support (0.36); severe multiple trauma (0.36); regional compliance (0.36); paediatric intensive care (0.36); individual level (0.34); chest examination (0.34); randomized trial (0.34); preterm lung (0.34); oscillatory volume (0.34); lung recruitability (0.34); absolute lung resistivity (0.34); early postoperative ventilation distribution (0.34); visual anatomical lung ct (0.34); severe ard (0.34); neonatal lung (0.34); noninvasive high-frequency ventilation (0.34); arm position (0.34); alveolar ventilation (0.34); overall ventilation inhomogeneities (0.32); term-born infant (0.32); chronic lung disease (0.32); following transition (0.32); noninvasive respiratory support (0.32); electron beam ct (0.32); preterm rabbit (0.32); different approaches (0.32); lung tissue properties (0.32); breathing lamb (0.32); bests positive end-expiratory pressure (0.32); local lung air content (0.32); supraglottic atomization (0.32); decremental positive end-expiratory pressure trial (0.32); receiving continuous positive airway pressure (0.32); quantifying ventilation distribution (0.32); gravity effect (0.31); virus infection (0.31); ongoing pathophysiological (0.31); mechanical function (0.31); histamine-provoked change (0.31); monitoring respiratory mechanics (0.31); birth-weight infant (0.31); therapeutic development (0.31); gradual tidal inflation (0.31) | 2004 |  |
| 3 | 72 | 0.827 | acute hypoxemic respiratory failure (136.11, 1.0E-4) | tissue properties (1.2); regional opening (1.2); open-lung approach (1.2); clinical usefulness (1.2); occult pendelluft (1.2); thorax eit (1.2); pig model (1.2); bedside monitoring (1.2); gravitational distribution (1.2); monitoring pulmonary perfusion (1.2); ventilation condition (1.2); lung abnormalities (1.2); surfactant treatment (1.2); perioperative assessment (1.2); paediatrics-a survey (1.2); clinical diagnosis (1.2); semi-siamese u-net (1.2); using lung density value (1.2); pulmonary perfusion (1.2); end-expiratory lung volume change (1.2); changing body position (1.2); patient (1.2); facilitating resolution (1.08); intratidal compliance profile analysis (1.08); breathing neonate (1.08); study protocol (1.08); lung function test (1.08); open abdominal surgery (1.08); individualized peep titration (1.08); of-bed elevation (1.08); bronchoscopic intervention (1.08); gravity-dependent lung ventilation (1.08); acute injury (1.08); pleural effusion drainage (1.08); protective mechanical ventilation (1.08); ventilation homogeneity (1.08); whither lung eit (1.08); neonatal piglet model (1.08); different body position (1.08); different bedside technique (1.08); lung recruitment maneuver (1.08); conventional lung (1.08); endotracheal tube position (1.08); interstitial lung diseases (1.08); regional ventilation inhomogeneity (1.08); long-term ventilator-induced lung injury ventilation (1); endotracheal intubation (1); lung-function test (1); blunt chest trauma (1); unilateral empyema (1); varying level (1); clinical scenario (1); ventilation scintigraphy (1); tidal breathing (1); regional filling characteristics (1); respiratory control (1); long-term porcine model (1); pressure support (1); severe multiple trauma (1); regional compliance (1); paediatric intensive care (1); individual level (0.95); chest examination (0.95); randomized trial (0.95); preterm lung (0.95); oscillatory volume (0.95); lung recruitability (0.95); absolute lung resistivity (0.95); early postoperative ventilation distribution (0.95); visual anatomical lung ct (0.95); severe ard (0.95); neonatal lung (0.95); noninvasive high-frequency ventilation (0.95); arm position (0.95); alveolar ventilation (0.95); bests positive end-expiratory pressure (0.91); decremental positive end-expiratory pressure trial (0.91); following transition (0.91); noninvasive respiratory support (0.91); electron beam ct (0.91); preterm rabbit (0.91); overall ventilation inhomogeneities (0.91); different approaches (0.91); lung tissue properties (0.91); breathing lamb (0.91); chronic lung disease (0.91); local lung air content (0.91); supraglottic atomization (0.91); term-born infant (0.91); receiving continuous positive airway pressure (0.91); quantifying ventilation distribution (0.91); monitoring respiratory mechanics (0.88); gravity effect (0.88); virus infection (0.88); ongoing pathophysiological (0.88); histamine-provoked change (0.88); birth-weight infant (0.88); therapeutic development (0.88); gradual tidal inflation (0.88); individualized lung recruitment strategies (0.88) | 2015 |  |
| 4 | 66 | 0.774 | spontaneous effort (148.22, 1.0E-4) | regional opening (0.7); gravitational distribution (0.7); open-lung approach (0.69); clinical usefulness (0.69); occult pendelluft (0.69); thorax eit (0.69); pig model (0.69); bedside monitoring (0.69); tissue properties (0.69); monitoring pulmonary perfusion (0.69); ventilation condition (0.69); lung abnormalities (0.69); surfactant treatment (0.69); perioperative assessment (0.69); paediatrics-a survey (0.69); clinical diagnosis (0.69); semi-siamese u-net (0.69); using lung density value (0.69); pulmonary perfusion (0.69); end-expiratory lung volume change (0.69); changing body position (0.69); patient (0.69); facilitating resolution (0.62); intratidal compliance profile analysis (0.62); breathing neonate (0.62); study protocol (0.62); lung function test (0.62); open abdominal surgery (0.62); individualized peep titration (0.62); of-bed elevation (0.62); bronchoscopic intervention (0.62); gravity-dependent lung ventilation (0.62); acute injury (0.62); pleural effusion drainage (0.62); protective mechanical ventilation (0.62); ventilation homogeneity (0.62); whither lung eit (0.62); neonatal piglet model (0.62); different body position (0.62); different bedside technique (0.62); lung recruitment maneuver (0.62); conventional lung (0.62); endotracheal tube position (0.62); interstitial lung diseases (0.62); regional ventilation inhomogeneity (0.62); blunt chest trauma (0.57); long-term porcine model (0.57); severe multiple trauma (0.57); endotracheal intubation (0.57); lung-function test (0.57); unilateral empyema (0.57); varying level (0.57); clinical scenario (0.57); long-term ventilator-induced lung injury ventilation (0.57); ventilation scintigraphy (0.57); tidal breathing (0.57); regional filling characteristics (0.57); respiratory control (0.57); pressure support (0.57); regional compliance (0.57); paediatric intensive care (0.57); individual level (0.54); chest examination (0.54); randomized trial (0.54); preterm lung (0.54); oscillatory volume (0.54); lung recruitability (0.54); absolute lung resistivity (0.54); early postoperative ventilation distribution (0.54); visual anatomical lung ct (0.54); severe ard (0.54); neonatal lung (0.54); noninvasive high-frequency ventilation (0.54); arm position (0.54); alveolar ventilation (0.54); following transition (0.52); noninvasive respiratory support (0.52); electron beam ct (0.52); preterm rabbit (0.52); overall ventilation inhomogeneities (0.52); different approaches (0.52); lung tissue properties (0.52); breathing lamb (0.52); bests positive end-expiratory pressure (0.52); chronic lung disease (0.52); local lung air content (0.52); supraglottic atomization (0.52); term-born infant (0.52); decremental positive end-expiratory pressure trial (0.52); receiving continuous positive airway pressure (0.52); quantifying ventilation distribution (0.52); gravity effect (0.5); virus infection (0.5); ongoing pathophysiological (0.5); histamine-provoked change (0.5); monitoring respiratory mechanics (0.5); birth-weight infant (0.5); therapeutic development (0.5); gradual tidal inflation (0.5); individualized lung recruitment strategies (0.5) | 2015 |  |
| 5 | 52 | 0.887 | increasing positive end-expiratory pressure  (136.21, 1.0E-4) | ventilation condition (0.34); perioperative assessment (0.34); changing body position (0.34); regional opening (0.34); open-lung approach (0.34); clinical usefulness (0.34); occult pendelluft (0.34); thorax eit (0.34); pig model (0.34); bedside monitoring (0.34); tissue properties (0.34); gravitational distribution (0.34); monitoring pulmonary perfusion (0.34); lung abnormalities (0.34); surfactant treatment (0.34); paediatrics-a survey (0.34); clinical diagnosis (0.34); semi-siamese u-net (0.34); using lung density value (0.34); pulmonary perfusion (0.34); end-expiratory lung volume change (0.34); patient (0.34); intratidal compliance profile analysis (0.3); individualized peep titration (0.3); facilitating resolution (0.3); breathing neonate (0.3); study protocol (0.3); lung function test (0.3); open abdominal surgery (0.3); of-bed elevation (0.3); bronchoscopic intervention (0.3); gravity-dependent lung ventilation (0.3); acute injury (0.3); pleural effusion drainage (0.3); protective mechanical ventilation (0.3); ventilation homogeneity (0.3); whither lung eit (0.3); neonatal piglet model (0.3); different body position (0.3); different bedside technique (0.3); lung recruitment maneuver (0.3); conventional lung (0.3); endotracheal tube position (0.3); interstitial lung diseases (0.3); regional ventilation inhomogeneity (0.3); regional compliance (0.28); endotracheal intubation (0.28); lung-function test (0.28); blunt chest trauma (0.28); unilateral empyema (0.28); varying level (0.28); clinical scenario (0.28); long-term ventilator-induced lung injury ventilation (0.28); ventilation scintigraphy (0.28); tidal breathing (0.28); regional filling characteristics (0.28); respiratory control (0.28); long-term porcine model (0.28); pressure support (0.28); severe multiple trauma (0.28); paediatric intensive care (0.28); lung recruitability (0.26); visual anatomical lung ct (0.26); individual level (0.26); chest examination (0.26); randomized trial (0.26); preterm lung (0.26); oscillatory volume (0.26); absolute lung resistivity (0.26); early postoperative ventilation distribution (0.26); severe ard (0.26); neonatal lung (0.26); noninvasive high-frequency ventilation (0.26); arm position (0.26); alveolar ventilation (0.26); following transition (0.24); noninvasive respiratory support (0.24); electron beam ct (0.24); preterm rabbit (0.24); overall ventilation inhomogeneities (0.24); different approaches (0.24); lung tissue properties (0.24); breathing lamb (0.24); bests positive end-expiratory pressure (0.24); chronic lung disease (0.24); local lung air content (0.24); supraglottic atomization (0.24); term-born infant (0.24); decremental positive end-expiratory pressure trial (0.24); receiving continuous positive airway pressure (0.24); quantifying ventilation distribution (0.24); mechanical function (0.23); assessing respiratory system (0.23); ongoing pathophysiological (0.23); therapeutic development (0.23); ventilatory support (0.23); gravity effect (0.23); virus infection (0.23); histamine-provoked change (0.23); monitoring respiratory mechanics (0.23) | 2012 |  |

**Supplement. Table2 Summary of EIT publications mentioned in the manuscript**

|  | Title | Publication Type | Topic | Author | Journal | Publication Year | Citations |
| --- | --- | --- | --- | --- | --- | --- | --- |
| 1 | Detection of local lung air content by electrical impedance tomography compared with electron beam CT | Animal study | Validation of the ability of EIT to detect local changes in air content with EBCT | Frerichs, I | J APPL PHYSIOL | 2002 | 138 |
| 2 | Regional ventilation by electrical impedance tomography: a comparison with ventilation scintigraphy in pigs. | Animal study | Validation of a good linear correlation between ventilation distribution measured by SPECT scan and EIT | Hinz, J | CHEST | 2003 | 92 |
| 3 | Imbalances in regional lung ventilation: a validation study on electrical impedance tomography. | Clinical study | Validation EIT could assessment of ventilation distribution during mechanical ventilation by comparison with CT | Victorino JA | AM J RESP CRIT CARE | 2004 | 196 |
| 4 | Noninvasive assessment of lung volume: respiratory inductance plethysmography and electrical impedance tomography | Review | Prospect of EIT as a noninvasive assessment of lung volume | Wolf, GK | CRIT CARE MED | 2005 | 27 |
| 5 | Lung volume recruitment after surfactant administration modifies spatial distribution of ventilation | Animal study | EIT assesses the effect of surfactant on the spatial distribution of mechanical ventilation | Frerichs, I | AM J RESP CRIT CARE | 2006 | 86 |
| 6 | Comparison of different methods to define regions of interest for evaluation of regional lung ventilation by EIT | Clinical study | Evaluation of EIT parameter ROI in 10 patients with controlled ventilation | Pulletz, Sven | PHYSIOL MEAS | 2006 | 78 |
| 7 | Assessment of regional lung recruitment and derecruitment during a PEEP study based on electrical impedance tomography. | Animal study | regional lung recruitment and derecruitment Assessment during a PEEP trial by eit | Meier, T | INTENS CARE MED | 2008 | 95 |
| 8 | Electrical impedance tomography compared with thoracic computed tomography during a slow inflation maneuver in experimental models of lung injury. | Animal study | EIT compared with CT during a slow inflation maneuver in different experimental models of lung injury | Wrigge, H | CRIT CARE MED | 2008 | 80 |
| 9 | Regional lung volume changes in children with acute respiratory distress syndrome during a derecruitment maneuver. | Clinical study | Regional lung volume changes during a derecruitment maneuver by eit in children with ARDS | Wolf, GK | CRIT CARE MED | 2007 | 29 |
| 10 | Bedside estimation of recruitable alveolar collapse and hyperdistension by electrical impedance tomography. | Clinical study | Validation of EIT for assessment of recurrent alveolar collapse and hyperdistension with CT | Costa, ELV | INTENS CARE MED | 2009 | 139 |
| 11 | Principles of electrical impedance tomography and its clinical application. | Review | Validation of the upcoming application of EIT in the clinic, particularly to optimize ventilatory therapy in patients with acute lung failure | Bodenstein, M | CRIT CARE MED | 2009 | 71 |
| 12 | Electrical impedance tomography compared to positron emission tomography for the measurement of regional lung ventilation: an experimental study. | Animal study | the measurement of regional lung ventilation with VTEIT and VPET | Richard, J | CRIT CARE | 2009 | 58 |
| 13 | Evaluation of an electrical impedance tomography-based Global Inhomogeneity Index for pulmonary ventilation distribution | Clinical study | Global Inhomogeneity Index from EIT for pulmonary ventilation distribution | Zhao, ZQ | INTENS CARE MED | 2009 | 98 |
| 14 | Electrical impedance tomography | Review | a review of EIT in monitoring the distribution of ventilation and alveolar lung collapse and hyperinflation and an outlook on ventilation / perfusion distribution | Costa, ELV | CURR OPIN CRIT CARE | 2009 | 139 |
| 15 | GREIT: a unified approach to 2D linear EIT reconstruction of lung images. | Review | a review of unified approach to 2D linear EIT reconstruction of lung images | Adler, A | PHYSIOL MEAS | 2009 | 109 |
| 16 | Regional tidal ventilation and compliance during a stepwise vital capacity manoeuvre. | Animal study | Regional tidal ventilation and compliance assesment of eit during a stepwise vital capacity manoeuvre | Dargaville, P | INTENS CARE MED | 2010 | 54 |
| 17 | PEEP titration guided by ventilation homogeneity: a feasibility study using electrical impedance tomography | Clinical study | PEEP titration guided by eit parameter ventilation homogeneity | Zhao, ZQ | CRIT CARE | 2010 | 64 |
| 18 | Electrical impedance tomography measured at two thoracic levels can visualize the ventilation distribution changes at the bedside during a decremental positive end-expiratory lung pressure study. | Clinical study | visualize the ventilation distribution changes during a decremental positive end-expiratory lung pressure trial by EIT measured at two positions | Bikker, IG | CRIT CARE | 2011 | 51 |
| 19 | Whither lung EIT: where are we, where do we want to go and what do we need to get there? | Review | Current status and future perspectives of lung EIT. How can existing data be used to help improve patient outcomes? | Adler, A | PHYSIOL MEAS | 2012 | 48 |
| 20 | Electrical impedance tomography: the holy grail of ventilation and perfusion monitoring? | Review | A review of the current use of EIT in ventilation perfusion imaging | Leonhardt, S | INTENS CARE MED | 2012 | 55 |
| 21 | Mechanical ventilation guided by electrical impedance tomography in experimental acute lung injury. | Animal study | Mechanical ventilation guided by conplaince measured by EIT in experimental acute lung injury | Wolf, GK | CRIT CARE MED | 2013 | 49 |
| 22 | Driving pressure and survival in the acute respiratory distress syndrome. | Clinical study | Among ventilatory variables, driving pressure was most strongly associated with survival compared with tidal volume and peep | Amato, M B P | NEW ENGL J MED | 2015 | 46 |
| 23 | Chest electrical impedance tomography examination, data analysis, terminology, clinical use and recommendations: consensus statement of the TRanslational EIT developmeNt stuDy group | Review | Review of all major work on chest EIT and recommendations for future development | Frerichs, I | THORAX | 2017 | 147 |
| 24 | Epidemiology, Patterns of Care, and Mortality for Patients with Acute Respiratory Distress Syndrome in Intensive Care Units in 50 Countries | Clinical study | Assessment of the incidence and prognosis and treatment status of ARDS in the ICU | Bellani, G | JAMA | 2016 | 30 |
| 25 | Variation of poorly ventilated lung units (silent spaces) measured by electrical impedance tomography to dynamically assess recruitment | Clinical study | poorly ventilated lung units measured by EIT to dynamically assess recruitment | Spadaro, S | CRIT CARE | 2018 | 23 |
| 26 | Electrical impedance tomography (EIT) in applications related to lung and ventilation: a review of experimental and clinical activities | Review | review summarizes the literature published over 15 years of EIT on lung monitoring | Frerichs, I | PHYSIOL MEAS | 2000 | 94 |
| 27 | Ventilation with lower tidal volumes as compared with traditional tidal volumes for acute lung injury and the acute respiratory distress syndrome. | Clinical study | Ventilation with lower tidal volumes compared with traditional tidal volumes for ARDS | Roy G B | NEW ENGL J MED | 2000 | 89 |
